# Preparation of Edible Films with *Lactobacillus plantarum* and Lactobionic Acid Produced by Sweet Whey Fermentation

**DOI:** 10.3390/membranes12020115

**Published:** 2022-01-19

**Authors:** Sara Sáez-Orviz, Ismael Marcet, Manuel Rendueles, Mario Díaz

**Affiliations:** Department of Chemical and Environmental Engineering, University of Oviedo, C/Julián Clavería 8, 33006 Oviedo, Spain; saezsara@uniovi.es (S.S.-O.); marcetismael@uniovi.es (I.M.); mariodiaz@uniovi.es (M.D.)

**Keywords:** deproteinised sweet whey, lactobionic acid, prebiotic, probiotic, edible film

## Abstract

Cheese whey, one of the most abundant by-products of the dairy industry, causes economic losses and pollution problems. In this study, deproteinised sweet whey was fermented by *Pseudomonas taetrolens* LMG 2336 to produce a prebiotic compound (lactobionic acid, LBA). Endotoxins produced by these microorganisms were successfully removed using microfiltration techniques, allowing the fermented whey permeate to be used in the food industry. The fermented whey permeate was used to develop prebiotic edible films by adding two different concentrations of gelatine (0.45 and 0.9 g gelatine g^−1^ LBA; LBA45 and LBA90). Furthermore, *Lactobacillus plantarum* CECT 9567 was added as a probiotic microorganism (LP45 and LP90), creating films containing both a prebiotic and a probiotic. The mechanical properties, water solubility, light transmittance, colour, and microstructure of the films were fully characterised. Additionally, the LBA and probiotic concentration in LP45 and LP90 were monitored under storage conditions. The strength and water solubility of the films were affected by the presence of LBA, and though all these films were homogeneous, they were slightly opaque. In LP45 and LP90, the presence of LBA as a prebiotic improved the viability of *L. plantarum* during cold storage, compared to the control. Therefore, these films could be used in the food industry to coat different foodstuffs to obtain functional products.

## 1. Introduction

Cheese whey is one of the most abundant by-products of the dairy industry. It is estimated that about 50% of the whey that is generated is disposed of directly into water systems, causing economic losses and pollution problems [1]. Thus, in the food industry, there is great interest in finding specific uses for the cheese whey generated during production processes in order to reduce environmental problems and economic costs. Cheese whey can be deproteinized, microfiltered, and ultrafiltered to generate concentrated whey protein [2] that can be used in different food products [1]. The liquid obtained after these processes is whey permeate. Whey permeate is composed mainly of lactose (5%), water (93%), and minerals (0.53%), with a minimal amount of proteins (0.85%) and fats (0.36%) [2]. Due to this poor composition, there are few ways to revalue this by-product of the dairy industry. One of them is the production by microbial fermentation of different compounds, such as oligosaccharides [2], lactic acid, and lactobionic acid (LBA) [3]. 

LBA has recently generated considerable attention in the food industry. This compound has many characteristics that make it potentially useful, such as its antioxidant [4], antimicrobial [5], and prebiotic properties [3], as it is resistant to the digestive environment and enzymes and can be metabolized by gastrointestinal microflora [6,7]. Although LBA has only been approved by the FDA for use in the form of the calcium salt (calcium lactobionate) [8,9], approval is under consideration by other food authorities and is expected in the short term. LBA is produced mostly by chemical synthesis [3], but it can also be produced by microbial fermentation. Obtaining LBA through biological synthesis has several advantages over chemical synthesis, as no undesirable products are generated, and no expensive metal catalysts are used [3]. By means of biological fermentation, numerous wastes from the food industry, such as cheese whey, can be used as substrates to obtain products with high added value. This fermentation can be carried out by several bacteria, such as *Zymomonas mobilis* or *Burkholderia cepacia* [3], but one of the most intensively studied is *Pseudomonas taetrolens* [10,11]. The LBA obtained has prebiotic capacity and therefore can be used to develop and improve foodstuffs and sustainable packaging materials.

Nowadays, conventional packaging materials developed from non-renewable origins are being replaced by more sustainable alternatives, such as edible casein [12], sodium alginate [13] or gelatine [14] films. The development of such films has attracted the attention of researchers and new types of materials have recently emerged [15]. Some of these new materials include whole grain flours (such as amaranth [16], quinoa [17], and chia [18]), fruit and vegetable residues (such as orange, lettuce, and carrot [19]), and root plants (such as starch from yam [20]). In addition, consumers are increasingly demanding food products that not only meet their nutritional demands but also have additional benefits, improving their health and reducing the risk of certain diseases. In this sense, the inclusion of bioactive compounds is a new trend in the development of packaging materials. Some of the bioactive compounds that can be added to films include antioxidants [21], antimicrobials [22], natural preservatives [23], and prebiotics. Thus, fermented whey permeate can be used to develop bioactive edible films with LBA as a prebiotic. Furthermore, if the prebiotic is combined with a probiotic, synbiotic packaging can be created. When both are combined, prebiotics can increase the survival and growth of probiotic microorganisms [24]. Regarding probiotics, the most commonly used belong to the genera *Bifidobacterium* and *Lactobacillus* [25]. 

Therefore, the aim of this study is to use a waste product from the food industry, such as sweet whey, as a substrate to develop an edible film with bioactive properties. For that purpose, a deproteinised sweet whey will be used as substrate and fermented by *P. taetrolens* LMG 2336, which converts the lactose in the substrate into LBA, resulting in a bioactive and high value-added product with prebiotic capacity. For use in the food industry, endotoxins produced by *P. taetrolens* (a Gram-negative microorganism) need to be removed, so the fermented whey will be microfiltrated. Gelatine is added to the fermented whey to form the protein matrix for the films. The bioactive edible films produced will be characterised regarding their mechanical and physical properties. In addition, *Lactobacillus plantarum* CECT 9567 will be added as a probiotic microorganism to analyse its viability inside the LBA edible films under storage conditions. 

## 2. Materials and Methods

### 2.1. Microorganism, Inoculum, Substrate, and Fermentation Conditions

#### 2.1.1. Microorganism and Growth Conditions

*Pseudomonas taetrolens* LMG 2336 (from the Belgian Coordinated Collection of Microorganisms, Ghent, Belgium) was used. The microorganism was inoculated on Nutrient Broth (NB, containing 1 g L^−1^ meat extract, 2 g L^−1^ yeast extract, 5 g L^−1^ peptone and 5 g L^−1^ NaCl, all from Sigma-Aldrich, Steinheim, Germany) agar (20 g L^−1^, VWR Chemicals, West Chester, PA, USA) plates and incubated at 30 °C for 48 h. A loopful from a fresh NB agar plate was inoculated in a 500 mL Erlenmeyer flask containing 100 mL of NB broth (Sigma-Aldrich) (ratio medium air 1:4). The inoculum was incubated in an orbital shaker (Model G25; New Brunswick Scientific Co., Edison, NJ, USA) at 250 rpm and 30 °C for 10 h. After this time, biomass was separated by centrifugation (10,000 rpm, 10 min) and was subsequently used as a bulk starter. 

#### 2.1.2. Sweet Whey Preparation

Deproteinised sweet whey (supplied by ILAS S.A., Asturias, Spain) with an initial concentration of approximately 200 g L^−1^ of lactose was diluted in distilled water to reach a concentration of approximately 40 g L^−1^ of lactose. The pH was adjusted to 6.5 by adding NaOH 10 N (Sigma-Aldrich). Then, it was sterilized, employing a tangential microfiltration unit equipped with a PVDF membrane-cassette (Pellicom 2 cassette, 0.5 m^2^ membrane area) with a pore size of 0.22 µm (Millipore, Billerica, MA, USA), a transmembrane pressure of 1.4 bar, and without recirculation. The permeate of the microfiltration was used as substrate in the fermentation.

#### 2.1.3. Culture in a Stirred Tank Bioreactor

Biomass of *P. taetrolens* LMG 2336 (obtained as explained in Section 2.1.1) was inoculated (at 10% (*v/v*)) in a 500 mL Erlenmeyer flask containing 100 mL of sterile deproteinised sweet whey, and it was incubated for 12 h at 30 °C, with an agitation of 250 rpm. After this time, the culture was again centrifuged (10,000 rpm, 10 min), and the biomass obtained was employed to inoculate the 2 L bioreactor (BioFlo 110; New Brunswick Scientific Co., Edison, NJ, USA), employing again 10% (*v/v*) of inoculum. The working volume of the bioreactor was 1 L with mechanical agitation. The operating parameters used were 30 °C, 350 rpm, and 1 Lpm aeration, following the conditions optimised by Alonso et al. (2012) [26] to maximize the production of LBA. Foaming was avoided by automatic addition of diluted (1:10) Y-30 emulsion (Sigma-Aldrich). The bioreactor was equipped with a pH-meter (Mettler Toledo, Greifensee, Switzerland). To maximize the LBA production, pH was left uncontrolled during the exponential growth phase and then with the automatic addition of 5M NaOH (Sigma-Aldrich) and was maintained at a value of 6.5 [26]. The bioreactor fermentation process lasted a total of 72 h. 

#### 2.1.4. Detection and Elimination of Bacterial Endotoxins

*P. taetrolens* LMG 2336, as other Gram-negative bacteria, produces endotoxins. These endotoxins must be eliminated so that the products obtained can be used in the food industry. To this end, after fermentation, the medium was centrifuged (10,000 rpm, 20 min) and tangentially microfiltered, employing the same equipment described in Section 2.1.2. The ToxinSensor™ Gel Clot kit (GenScript, Piscataway, NJ, USA) was used to detect the presence of endotoxins in the permeate, employing the semi-quantitative detection protocol (minimum detected concentration of endotoxins of 0.25 EU mL^−1^ (EU, endotoxin units)). 

### 2.2. Film Preparation

Four different types of film-forming solutions (Table 1) were prepared as follows. The fermented whey was tangentially microfiltered and the concentration of LBA in the permeate was determined as was stated in previous work [27]. Then, gelatine (Sigma-Aldrich) was added to the permeate at 0.45 and 0.9 g of gelatine per g of LBA, which represented an addition of 1.8 and 3.6 g of gelatine per 100 mL^−1^ of fermented whey permeate, and the resulting mixtures were heated at 40 °C until clear solutions were obtained, constituting the LBA45 and LBA90 film-forming solutions. These two different concentrations of gelatine were selected according to previous tests and in order to evaluate the effect of the concentration of this protein on the properties of the films obtained.

In addition, in order to produce synbiotic films, *Lactobacillus plantarum* CECT 9567 (from the Spanish Type Culture Collection, Valencia, Spain) was added to the LBA45 and LBA90 film-forming solutions until a concentration of 8 log_10_ CFU mL^−1^ was reached, obtaining the LP45 and LP90 film-forming solutions. *L. plantarum* was previously grown in MRS Broth (de Man, Rogosa and Sharpe, Sigma-Aldrich) in aerobic conditions, at 30 °C for 24 h. 

To obtain the dried films, 20 mL of each film-forming solution was cast in a Petri dish of 90 mm of diameter and dried in an oven at 40 °C for 24 h. Finally, the films could be removed from the Petri dishes without being sticky or brittle. Two controls prepared only with gelatine and distilled water maintaining the same ratio as in LBA films (1.8 and 3.6 g gelatine 100 mL^−1^) were also prepared.

### 2.3. Film Characterization

#### 2.3.1. Composition of the Film 

To check the amount of LBA in the films, high-performance liquid chromatography (HPLC) was employed, according to Sáez-Orviz et al. (2019) [27]. First, 0.5 g of each film sample was dissolved in 1 mL of distilled water and was filtered (0.22 µm, Whatman, Sigma-Aldrich) before HPLC analysis. The chromatography equipment used (Agilent 1200, Agilent Technologies Inc., Santa Clara, CA, USA) was fitted with a Coregel ION 300 column (Teknocroma, Barcelona, Spain) coupled to a refractive index detector (at a temperature of 40 °C). Sulphuric acid solution (0.450 mM L^−1^, pH 3.1) was used as a mobile phase with a flow rate of 0.3 mL min^−1^ and a column temperature of 75 °C. Data acquisition and analysis were performed with ChemStation software (Agilent).

#### 2.3.2. Thickness and Mechanical Properties

The thickness of the films was measured using a digital micrometer (Mitutoyo, Kawasaki, Japan). The analysis was performed at ten different points, both in the centre and outer parts of the films. Film thickness is reported as the average of these measurements. 

Mechanical characterisation was performed using a TA.XTplus Texture Analyzer (Stable Systems, Godalming, Surrey, UK). Films were cut into squares and were submitted to a penetration test, at room temperature, employing a spherical SMS P/5S probe with a test speed of 2.0 mm s^−1^ and a 5 kg load cell. Results are expressed in terms of puncture strength (PS) and puncture deformation (PD). Both parameters were calculated according to the following equations:

(1) PS=Fm/Th

(2) $$\mathrm{PD}=\left(\sqrt{D^{2}+R^{2}}-R\right)/R\;$$

Fm is the maximum force applied before the breakage of the film (N), Th is the film thickness (mm), D is the distance covered by the probe while it is in contact with the film until the film is broken (mm), and R is the radius of the orifice in the plates (mm). Experiments were carried out in triplicate and reported results correspond to the mean value.

#### 2.3.3. Water Solubility (WS)

Water solubility (WS) was measured as follows. Films were cut into circumferences of 40 mm diameter and were immersed in 20 mL of distilled water with 2% (*w/w*) Tris-HCl pH 7.0 (Sigma-Aldrich). Samples were kept for 24 h at room temperature. After this time, the mixture was filtered using a vacuum pump and Whatman no 1 paper (Sigma-Aldrich) to recover the insolubilized pieces of films, and they were dried in an oven at 105 °C for 24 h. WS was calculated as follows [28]:

(3) WS(%)=(m1−m2)/m1×100 

where m1 is the weight (g) of the dry films, and m2 is the weight (g) of the solubilized and dry films. Experiments were carried out in triplicate and the reported results correspond to the mean value.

#### 2.3.4. Optical Transmittance and Transparency Index

Optical transmittance and transparency of the films were measured as follows. Rectangular pieces of each film were cut and placed in a spectrophotometer test cell. Wavelengths from 200 to 800 nm were tested with a Helios gamma spectrophotometer (Thermo Fischer Scientific, Waltham, MA, USA). An empty test cell was used as reference. Transparency of the films was calculated as follows [29]:

(4) $$\mathrm{Transparency}=A_{600}/x\;$$

where A_600_ is the film absorbance at 600 nm and x is the thickness of the film (mm). 

#### 2.3.5. Colour Properties

The colour of the film samples was measured employing a Lovibond^®^ LC100 Spectrocolorimeter (Tintometer^®^ Group, Lovibond house, Amesbury, UK). Three parameters were measured: L* (lightness/brightness), a* (redness/greenness), and b* (yellowness). A white standard plate was tested. The total colour difference (ΔE) was calculated as follows [30]:

(5) $$\Delta E=\sqrt{{\left(\Delta L^{*}\;\right)}^{2}+{\left(\Delta a^{*}\;\right)}^{2}+{\left(\Delta b^{*}\;\right)}^{2}\;}\;$$

where ΔL*, Δa*, and Δb* are the difference between the colour of the white standard plate (L* = 94.00, a* = 0.8 and b* =1.1) and film samples. The b* and L* parameters are linked to yellowness index (*YI*) according to the following equation [31]:

(6) $$YI=\left(142.86\times b^{*}\right)/L^{*}\;$$

Experiments were carried out in triplicate, and the reported results correspond to the mean value. 

#### 2.3.6. Scanning Electron Microscopy (SEM)

Surface and cross-sectional film morphology was observed, employing scanning electron microscopy (SEM) (JSM-6610LV, JEOL, Peabody, MA, USA). Film samples were freeze-dried (0.1 mBar, −70 °C for 24 h) and cut into squares with a surgical blade. The film squares were mounted around stubs and coated with gold. In order to obtain micrographs of the entire cross-section, and due to the difference in thickness of the samples tested, the magnification values used varied from 2000× to 250× depending on the composition of the film assessed. 

#### 2.3.7. Viability of *Lactobacillus plantarum* CECT 9567 and Lactobionic Acid Evolution inside the Films under Storage Conditions

The probiotic growth and LBA concentration were followed for 15 days. In addition to viability in LP45 and LP90 films, two control experiments without LBA were also carried out in C45 and C90 films additivated with *L. plantarum* (C45LP and C90LP), with the aim to analyse the effect of LBA on the viability of the microorganism. Samples were taken at 0, 1, 3, 8, 10, 13, and 15 days. For both measurements, 0.1 g of LP45 and LP90 film samples were placed in a Stomacher^TM^ bag (Seward, UK) with 1 mL of NaCl 0.7% (*w/v*) (Sigma-Aldrich) and were homogenized with a Stomacher^TM^ device (Seward, UK) at medium speed for 60 s to dissolve the film. Microbial growth was analysed by preparing serial dilutions (1:10) and incubating on MRS agar plates for 48 h at 30 °C. The results were expressed in log_10_ CFU g^−1^. LBA concentration was also measured as explained in Section 2.3.1. In both cases, each sample was performed in triplicate. 

### 2.4. Statistical Analysis

Analysis of variance (ANOVA) was applied. Fisher’s Least Significant Difference (LSD) procedure was performed to determine significant differences between the data and a level of *p* < 0.05 was considered significant. Statgraphics 18^®^ Centurion software was used for the analysis. 

## 3. Results and Discussion

### 3.1. Analysis of Fermented Whey Composition and Removal of Endotoxins

The presence of endotoxins was checked after fermentation in the bioreactor. The result with the ToxinSensor™ Gel Clot kit was positive in accordance with the manufacturer’s instructions, indicating the presence of a concentration of endotoxins higher than 0.25 EU mL^−1^ in the fermented whey. After centrifugation and tangential microfiltration of the fermented whey, the endotoxin assay was performed again in the permeate fraction. The results were negative, so the concentration of endotoxins was lower than 0.25 EU mL^−1^. As far as is known, there is no limit available for endotoxin present in orally administered products [32]. Despite this, the concentration is far below the values required in the pharmaceutical industry (5 EU per kg of body mass) [32]. Therefore, the product obtained with this microfiltered fermented whey can be considered safe. Endotoxins tend to form globules or aggregates in aqueous solutions [33]. The globules can have a variety of shapes [34], and they are larger and bigger in the presence of different ions, such as Ca^2+^, Mg^2+^, and Na^+^ [33]. Individual endotoxin molecules have a molecular weight between 10 and 20 kDa, but these larger aggregates can have a molecular weight as high as 1 MDa [33]. The sweet whey permeate has a considerable concentration of calcium [35], and the presence of Ca^2+^ leads to a greater aggregation of the endotoxins, thus facilitating their retention in the tangential microfiltration process, which allows their removal to an acceptable concentration. This is an important point for the potential use of LBA as a food component that has been produced from a by-product of the food industry, as none of the research on whey fermentation to produce LBA by *P. taetrolens* has investigated this problem [11,36,37,38]. 

Regarding the composition of fermented whey, only lactose and LBA were measured, as the permeate has a very low concentration of fat (0.36%) and proteins (0.85%) [2]. LBA concentration after fermentation was 44.69 ± 5.84 g L^−1^, and the lactose concentration was 7.36 ± 0.57 g L^−1^. After the microfiltration process to remove endotoxins, the concentration of both compounds was again analysed, yielding 40.92 ± 1.09 and 6.32 ± 0.84 g L^−1^ for LBA and lactose, respectively. The microfiltered fermented whey was diluted with water to a concentration of 15 g L^−1^ of LBA. There is currently no established legal concentration for human consumption [8], but the LBA concentration used to make the films is much lower than the values that would cause effects similar to lactose intolerance (24 g LBA per day [39]). 

### 3.2. Characterization of the Films

#### 3.2.1. Thickness and Mechanical Properties of the Films

The thickness of the edible films is shown in Table 2. Four groups were distinguished with significant differences (*p* < 0.05): C45, C90, LBA45-LP45, and LBA90-LP90. The presence of LBA made the films thicker compared to the control films. This may be because LBA molecules are very hygroscopic [4] and have a higher capacity to retain water, making the films thicker. Furthermore, the more gelatine added to LBA films, the thicker they became. Regarding the presence of probiotics, this did not influence the thickness of the films.

The presence of LBA affected the mechanical properties of the films (Table 2). PS values obtained in films with the same amount of gelatine (LBA90 and LP90) were lower than C90 and similar to the control with less amount of gelatine (C45). LBA45 and LP45 showed the lowest PS values, so the presence of LBA in the edible films made them less resistant. LBA molecules are very hygroscopic (54 g mol^−1^) in comparison with other agents such as glycerol (33 g mol^−1^) or sorbitol (14 g mol^−1^) [40] and have a greater capacity to retain water [4]. Due to these characteristics, the presence of LBA makes the films thicker but mechanically weaker.

In the case of PD, significant differences were found when the probiotic was added, since LP45 and LP90 showed the highest values (Table 2), indicating that the addition of *L. plantarum* made the films stickier. This can be explained by the fact that the microbial mass increases the stickiness of the film-forming solution and thus of the films themselves, resulting in higher PD values. Other authors also observed that the addition of microorganisms affected the elongation break parameter [41,42]. 

#### 3.2.2. Water Solubility (WS)

The WS of LBA and LP edible films at room temperature was higher than of the gelatine control films (Table 2). Due to the chemical characteristics of LBA, it has a high interaction with water, which makes it easily soluble in aqueous media. In a recent study, 400 mg mL^−1^ of LBA were completely dissolved at room temperature [4]. Despite their high solubility, WS values are lower than for other films such as casein films [12], soybean polysaccharides [43], or sodium alginate [13]. It was also observed that the presence of probiotic microorganisms did not alter the solubility of the films. This result was also observed by other authors [41,44].

#### 3.2.3. Light Transmittance and Transparency Index

**Figure 1:**
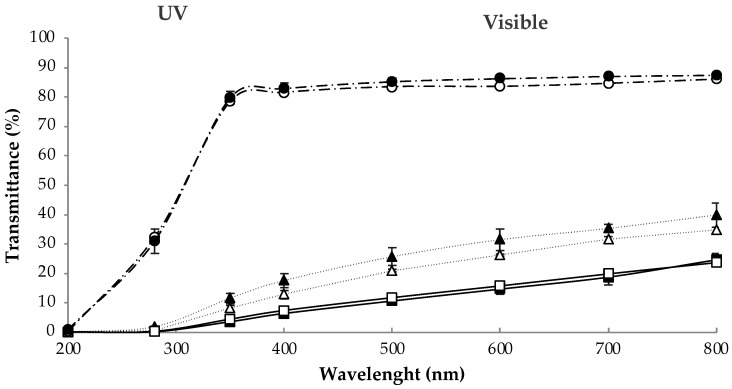
Light transmittance (%) of the edible films at different wavelengths (200–800 nm). (●) C90, (○) C45, (▲) LBA45, (△) LP45, (■) LBA90, and (□) LP90. Statistically (*p* < 0.05), three different groups were observed: control samples (C45, C90), LBA45-LP45, and LBA90-LP90.

The light transmittance of the edible films was analysed over a wavelength range between 200 and 800 nm (Figure 1). Low transmission in the UV range is a desirable property for food packaging material, since 98% of UV radiation (315 to 400 nm) reaches the earth [45], favouring the oxidation of foods with a high lipid content [12]. For the UVC (100–280 nm) and UVB (280–320 nm) regions, the transmittance levels of all the film samples were very low. In the UVA (320–400 nm) range, there was an increase in the optical transmittance only for the control samples (C45 and C90). 

For wavelengths in the visible region, the optical transmittance of the LBA edible films was very low compared with the control edible films (Figure 1). The maximum transmittance values, reached at 800 nm, were 39.97%, 34.65%, 24.60%, and 23.67% for LBA45, LP45, LBA90, and LP90 edible film samples, respectively. There were no significant differences in transmittance values between the controls with the two different amounts of gelatine (C45 and C90), but there were significant differences between the LBA and LP edible film samples. Regarding the differences between the control and the LBA and LP edible films, the results obtained were consistent with the transparency index (Table 2), since control films were more transparent than the LBA and LP films. The difference between control and LBA-LP films may be due to their high content in LBA, which may increase its interactions with the proteins as the film-forming solution dries and the water evaporates, changing the conformation of the proteins and aggregating in different ways when the films are formed. Regarding the LBA and LP films, LBA90 and LP90 were the ones with the lowest transmittance values. This may be explained by the increase in thickness of the films with more gelatine (Table 1), which could cause a decrease in the transmittance values observed.

Besides that, the addition of probiotic microorganisms did not influence this parameter, as LBA45-LP45 and LBA90-LP90 groups did not show significant differences.

#### 3.2.4. Colour Properties

Colour properties are important in the development of food packaging, as the visual appearance of food products is a key factor that directly influences consumer acceptability. The edible film colour properties are shown in Table 3. Significant differences (*p* < 0.05) were found in all parameters measured (L*, a*, b*, ΔE and *YI*), but only between control and LBA edible films samples, regardless of whether they had probiotic microorganisms or not. All LBA films were slightly yellowish, as the *YI* parameter showed (Table 3). This yellowish hue may be due to the natural colour of the sweet whey permeate [2], which remains intact after microbial fermentation. 

**Figure 2:**
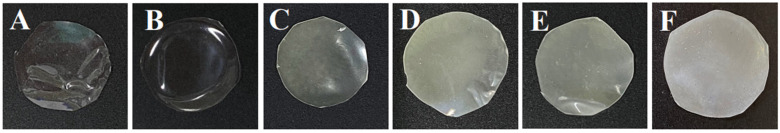
Visual appearance of the edible films. (**A**) C45, (**B**) C90, (**C**) LBA45, (**D**) LBA90, (**E**) LP45, and (**F**) LP90 edible films.

These data are reinforced by the visual appearance of the films (Figure 2), where it was observed that the controls were completely transparent and colourless while the edible LBA films had a more opaque and yellower appearance. Despite these differences, edible films were homogeneous in all cases.

#### 3.2.5. Scanning Electron Microscopy 

**Figure 3:**
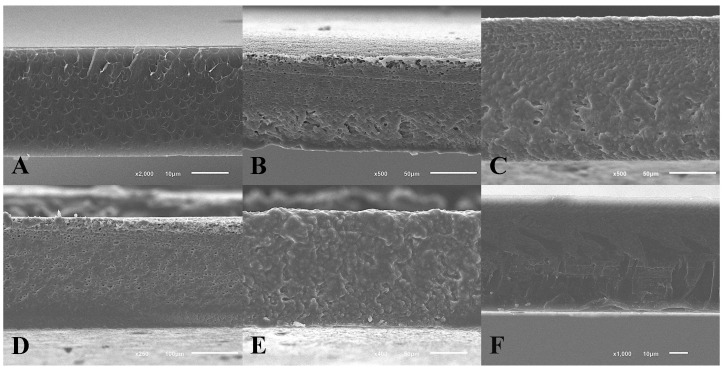
SEM images of the edible film cross-sections. (**A**) C45 (2000×); (**B**) C90 (500×); (**C**) LBA45 (500×); (**D**) LBA90 (250×); (**E**) LP45 (400×); and (**F**) LP90 (1000×).

The surface microstructure of all tested films was homogeneous, without pores and with no difference between them (data not shown). In order to evaluate the degree of compaction of the film matrix, micrographs of the cross section of the edible films were also carried out (Figure 3). The presence of LBA and *L. plantarum* did not produce noticeable changes in the microstructure of the films. However, the percentage of gelatine did. LBA90 and LP90 edible films showed a more compact structure with less agglomerated proteins than LBA45 and LP45 films. This agrees with the results obtained for the PS values (Table 2), where films with 60% gelatine obtained better values than those with 30% gelatine. In the case of the controls, protein agglomerations were observed in both cases.

#### 3.2.6. Viability of *Lactobacillus plantarum* CECT 9567 and Lactobionic Acid Evolution inside the Films under Storage Conditions

**Figure 4:**
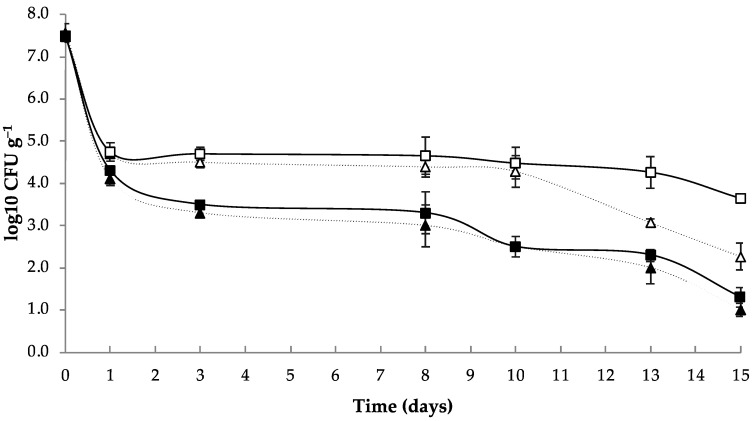
Growth of *L. plantarum* in LP45 (△), LP90 (□), C45LP (▲), and C90LP (■) edible films over 15 days of storage. The amount of microorganism is represented as log_10_ CFU per g of film. C45LP and C90LP did not show significant differences between them, whereas significant differences were found between LBA45 and LBA90 from day 13 to 15. Experiments were performed in triplicate and reported results correspond to the mean value.

The growth of the probiotic inside LP edible films is shown in Figure 4. In all cases, there was a rapid decrease in the *L. plantarum* concentration between the initial time and the first day, with a reduction of three logarithmic units in the samples. This result indicated that the process conditions have had an impact on bacterial survival. *L. plantarum* was transferred from the culture medium to the nutrient-poor film-forming solution and was dried at 40 °C, which may have caused a rapid loss of moisture and thus a reduction in the viability of the microorganisms [46]. In the case of the C45LP and C90LP films, the viability was much lower due to the presence of only gelatine in the composition of the film. The presence of LBA improved the viability of *L. plantarum*, as shown in Figure 4. In films LP45 and LP90, after the initial decrease, a concentration of approximately 4.5 log_10_ CFU g^−1^ of film remained constant until day 13, when the microbial concentration decreased again, reaching values of 2.30 and 3.64 log_10_ CFU g^−1^ of LP45 and LP90 film, respectively. This may be due to the lack of nutrients that can be used as substrate for the maintenance and growth of the probiotic bacteria. As other authors also point out, the storage temperature (4 °C) could also affect probiotic survival [47,48], as the optimum temperature for *L. plantarum* growth is 30 °C. 

**Figure 5:**
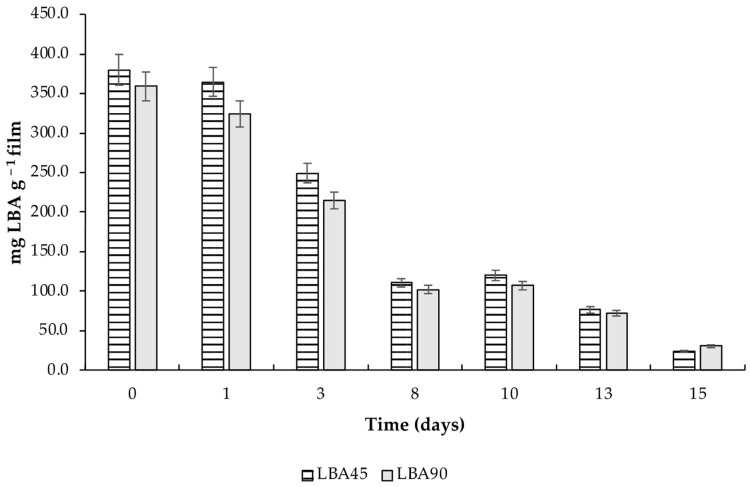
Change in LBA concentration in LP45 and LP90 edible films over 15 days of storage. LBA concentration is represented as mg of LBA per g of film. There were no significant differences (*p* > 0.05). Experiments were performed in triplicate and reported results correspond to the mean value.

The probiotic viability results agreed with the analysis of the concentration of LBA in the edible films during 15 days of storage (Figure 5). It was observed that the concentration of LBA decreased. On day 13, there was a reduction in LBA concentration of 79.0% for LBA45 films and 79.97% for LBA90 films, so the probiotic was using the prebiotic compound as substrate. At the final point, the reduction in prebiotic concentration was 93.82% and 91.48% for LBA45 and LBA90, respectively. Thus, the decrease in probiotic concentration during the last days is due to the absence of nutrients in the film. In consequence, the presence of LBA favoured the viability of *L. plantarum* during cold storage. Similar studies using LBA as a prebiotic compound have shown an enhancement in the viability of probiotic microorganisms [29,49,50]. The improvement of microbial viability during cold storage in the presence of prebiotics has also been found with other compounds such as inulin [51] and fructo-oligosaccharides [52]. Therefore, films developed from a by-product of the food industry could be used, in the same industry, to coat dairy products, such as cheese, thus providing them not only with protection, but also with the added value of a prebiotic and probiotic microorganism pairing, which would make it possible to obtain a functional food product.

## 4. Conclusions

Edible films enriched in LBA employing fermented whey and gelatine as protein matrix were successfully developed and characterised. The deproteinised sweet whey was fermented with *P. taetrolens* and endotoxins were successfully removed after the microfiltration process, allowing the safe use of the permeate in the food sector. Control, LBA45, LP45, LBA90, and LP90 edible films showed adequate mechanical properties, although the PS parameter was influenced by the presence of LBA and the PD by the presence of the probiotic microorganism. In addition, as was expected, films with a higher content in gelatine also showed greater strength. The edible films showed medium water solubility, largely because the chemical characteristics of LBA made them very soluble in water. Films were homogeneous and slightly opaque, as LBA somehow interacts with the added gelatine itself. Regarding the addition of *L. plantarum* as a probiotic microorganism, results for LP45 and LP90 were similar, indicating that the amount of gelatine did not influence the viability of the bacteria. However, the presence of LBA did have an influence, as this prebiotic compound favoured the maintenance of *L. plantarum* viability during cold storage by being consumed as substrate. A future line of research would be to study the behaviour of these films in food products, as well as the possible protective capacity for the probiotics of the films themselves in in vitro digestion tests.

## Figures and Tables

**Table 1:** Composition of the different types of film-forming solutions prepared.

Samples	Gelatine (g 100 mL^−1^ Film-Forming Solution)	LBA (g 100 mL^−1^ Film-Forming Solution)	*Lactobacillus plantarum* CECT 9567
Control (C45)	1.8	-	-
Control (C90)	3.6	-	-
LBA45	1.8	~4	-
LBA90	3.6	~4	-
LP45	1.8	~4	8 log_10_ CFU mL^−1^
LP90	3.6	~4	8 log_10_ CFU mL^−1^

**Table 2:** Puncture strength (PS), puncture deformation (PD), thickness, water solubility (WS), and transparency index of the four edible film samples. Different letters in the same column indicate significant differences (*p* < 0.05).

Sample	PS (N mm^−1^)	PD (%)	Thickness (µm)	WS (%)	Transparency Index
C45	82.0 ± 12.2 ^a^	7.8 ± 2.4 ^a^	50.67 ± 7.15 ^a^	20.4 ± 0.9 ^a^	1.05 ± 0.15 ^a^
C90	130.3 ± 13.2 ^b^	13.7 ± 7.9 ^b^	75.50 ± 5.3 ^b^	21.1 ± 2.3 ^a^	1.68 ± 0.07 ^a^
LBA45	31.9 ± 3.6 ^c^	11.8 ± 2.7 ^b^	165.4 ± 38.5 ^c^	63.5 ± 3.4 ^b^	3.18 ± 0.29 ^b^
LBA90	82.5 ± 11.3 ^a^	12.6 ± 4.1 ^b^	212.0 ± 36.9 ^d^	66.8 ± 4.0 ^b^	3.45 ± 0.24 ^c^
LP45	27.7 ± 2.0 ^c^	28.5 ± 2.8 ^c^	194.1 ± 22.9 ^c^	60.8 ± 3.5 ^b^	2.87 ± 0.03 ^b^
LP90	73.7 ± 11.4 ^a^	33.8 ± 3.5 ^c^	230.3 ± 28.9 ^d^	61.0 ± 4.6 ^b^	3.48 ± 0.07 ^c^

**Table 3:** Colour of the edible film samples. L* (lightness/brightness), a* (redness/greenness), b* (yellow/blueness), ΔE (total colour difference), *YI* (yellowness index), and opacity of the edible film samples. Different letters in the same column indicate significant differences (*p* < 0.05).

Sample	L*	a*	b*	Δ*E*	*YI*
C45	92.1 ± 0.6 ^a^	0.5 ± 0.2 ^a^	1.7 ± 0.6 ^a^	2.3 ^a^	2.69 ^a^
C90	92.3 ± 0.2 ^a^	0.17 ± 0.3 ^a^	1.2 ± 0.8 ^a^	2.1 ^a^	1.79 ^a^
LBA45	93.9 ± 0.7 ^b^	0.5 ± 0.2 ^b^	7.3 ± 0.7 ^b^	6.3 ^b^	11.16 ^b^
LBA90	92.6 ± 0.9 ^b^	0.3 ± 0.1 ^b^	7.6 ± 0.4 ^b^	6.8 ^b^	11.98 ^b^
LP45	94.0 ± 0.5 ^b^	0.4 ± 0.2 ^b^	7.9 ± 0.5 ^b^	6.9 ^b^	12.01 ^b^
LP90	92.5 ± 1.0 ^b^	0.4 ± 0.1 ^b^	7.6 ± 0.4 ^b^	6.7 ^b^	11.74 ^b^
